# Glioblastoma as Developmental Stress Boundary Displacement: Insect Embryonic Cells as Quantitative Reference Platforms for Mitochondrial Adaptation—A Conceptual Framework

**DOI:** 10.3390/cells15151352

**Published:** 2026-07-28

**Authors:** Wojciech Rzeski

**Affiliations:** Department of Functional Anatomy and Cytobiology, Institute of Biological Sciences, Maria Curie-Skłodowska University, Akademicka 19, 20-033 Lublin, Poland; wojciech.rzeski@mail.umcs.pl

**Keywords:** mitochondrial stress adaptation, developmental stress boundary, glioblastoma, insect embryonic systems, redox homeostasis, ferroptosis, proteostasis, metabolic plasticity

## Abstract

Glioblastoma (GBM) remains one of the most therapy-resistant human malignancies, yet the physiological limits of mitochondrial stress adaptation against which its remarkable resilience might be interpreted remain poorly defined. This opinion article proposes the developmental stress boundary framework, a hypothesis-generating model in which insect embryonic cells, particularly those of *Drosophila melanogaster*, serve as an experimentally tractable, non-malignant reference platform for quantitative mapping of mitochondrial stress tolerance. Within this framework, GBM stress tolerance may be interpreted not as the product of fundamentally novel adaptive mechanisms, but as a pathological displacement and long-term stabilization of conserved mitochondrial programs transiently engaged during normal embryonic development. The framework is intended to complement, rather than replace, existing models of glioblastoma biology. The proposed framework integrates four interconnected dimensions of mitochondrial adaptation—redox homeostasis, lipid metabolism and ferroptosis-like resistance, proteostasis, and metabolic flexibility—into a multidimensional physiological stress boundary that defines the limits of reversible cellular adaptation. Comparative evidence from developmental biology and glioblastoma research is consistent with the possibility that malignant cells expand these conserved adaptive programs beyond their normal physiological constraints, although this relationship has not yet been directly tested. The framework generates four testable predictions: (i) stress–response curves in GBM may be right-shifted relative to embryonic baselines while preserving their overall architecture; (ii) mesenchymal-like GBM states may exhibit the greatest displacement of stress tolerance; (iii) effective therapeutic strategies may require simultaneous compression of multiple adaptive dimensions to prevent compensatory responses; and (iv) pharmacodynamic biomarkers, including lipid peroxidation products and GSSG/GSH ratios, could provide early indicators of declining stress-buffering capacity during treatment. Establishing quantitative developmental baselines using insect embryonic systems, alongside complementary reference points such as tissue regeneration, may provide a conceptual and experimental basis for interpreting mitochondrial stress adaptation in glioblastoma. Rather than proposing a new explanatory model of glioblastoma biology, this framework seeks to provide a quantitative developmental reference against which mitochondrial stress adaptation in glioblastoma can be experimentally interpreted, tested, and, where necessary, revised.

## 1. Introduction

How far beyond physiological developmental limits does glioblastoma extend its stress tolerance—and can those limits be quantitatively defined? Current models of cancer plasticity describe developmental reactivation, metabolic rewiring, and stress adaptation, but none explicitly addresses how far these processes extend beyond their physiological developmental range or where their adaptive limits lie. Embryonic development requires cells to undergo rapid proliferation, differentiation, migration, and metabolic remodeling while preserving viability and structural integrity. Glioblastoma (GBM), the most common malignant primary brain tumor in adults, exhibits remarkable tolerance to oxidative, metabolic, and proteostatic stress. Despite standard-of-care therapy, median overall survival remains approximately 15 months [1]. Yet developmental transitions do not proceed without constraints. Cellular adaptation operates within physiological limits, beyond which oxidative damage, lipid instability, energetic collapse, and proteotoxic failure become irreversible, thereby defining the boundary between productive plasticity and catastrophic dysfunction. Here, the developmental stress boundary framework positions insect embryonic cells as one quantitative reference system for defining the physiological limits of mitochondrial stress adaptation and determining whether those limits are pathologically displaced in glioblastoma. This framework is intended to complement, rather than replace, existing genetic, metabolic, and developmental models of glioblastoma biology.

Although numerous models describe glioblastoma plasticity in terms of genetic, metabolic, and developmental reprogramming, none explicitly defines the physiological limits within which adaptive mitochondrial responses operate during normal development. This conceptual gap motivates the framework proposed here.

Although metabolic rewiring, stress tolerance, and developmental program reactivation are shared by many aggressive malignancies, GBM combines several features that make it a particularly informative test case for the proposed mitochondrial stress-boundary framework. GBM arises from neural lineages and displays well-characterized, interconvertible cellular states that retain identifiable relationships to developmental transcriptional programs [2,3]. GBM also develops within the metabolically constrained brain microenvironment, where heterogeneous perfusion, hypoxia, and fluctuating nutrient availability may select for cells with an expanded capacity to tolerate stress. Its near-uniform lethality despite aggressive multimodal therapy [1] underscores the clinical importance of adaptive resistance mechanisms, among which stress tolerance may be a major contributor. These features do not make GBM biologically unique among cancers, but they render it a clinically consequential and conceptually informative system in which the proposed framework can be examined. Extending the framework to other highly plastic malignancies, including leukemia, represents a natural direction for future research.

Mitochondria are central regulators of these constraints. Beyond energy production, they integrate redox signaling, biosynthetic flux, lipid metabolism, and stress-response pathways that collectively determine whether cells maintain reversible adaptation or progress toward dysfunction [4,5,6,7,8,9,10]. Moderate mitochondrial reactive oxygen species (ROS) signaling contributes to developmental transitions and stem cell regulation [4,6], whereas sustained oxidative stress compromises viability and promotes damage accumulation [5,7]. Similarly, shifts between glycolysis and oxidative phosphorylation support developmental plasticity but remain tightly regulated in both timing and magnitude [8,9,10]. Within the proposed framework, developmental plasticity can be viewed as adaptive mitochondrial regulation operating within physiological limits. This boundary does not represent a single threshold. Rather, it reflects a dynamic, multidimensional constraint defined by the interaction of oxidative balance, metabolic flexibility, lipid stability, and proteostatic capacity. Exceeding these limits is expected to result in irreversible cellular dysfunction rather than productive developmental adaptation. The framework therefore interprets GBM stress tolerance not as arising from an entirely distinct adaptive architecture, but as the pathological displacement and stabilization of mitochondrial programs transiently engaged during normal development. This interpretation captures conserved stress-adaptation modules rather than the full causal complexity of GBM, which also encompasses oncogenic rewiring, chromatin remodeling, and microenvironmental interactions lying outside its scope. The following sections develop this conceptual framework, summarize the evidence underlying its biological rationale, and outline the experimentally testable predictions it generates.

## 2. Mitochondria Integrate Redox, Lipid Metabolism and Proteostasis

During embryogenesis, mitochondrial activity coordinates redox balance, lipid metabolism, biosynthetic flux, and proteostatic demand [4,5,6,7,8,9,10,11,12,13,14,15], and these processes are tightly interconnected rather than functionally isolated.

Mitochondrial ROS participate in signaling pathways regulating differentiation and stem cell transitions [4,5,6,7,9,16], but increased oxidative flux also promotes lipid peroxidation, perturbs membrane integrity, and elevates protein-folding demand. Rapid developmental growth requires coordinated membrane biogenesis and lipid remodeling [11,12,17], while excessive lipid peroxidation destabilizes membranes and can trigger ferroptotic collapse [18,19]. Increased biosynthetic activity likewise imposes proteostatic pressure buffered through chaperone systems and unfolded protein responses in the ER and mitochondria [13,14,15].

**Figure 1:**
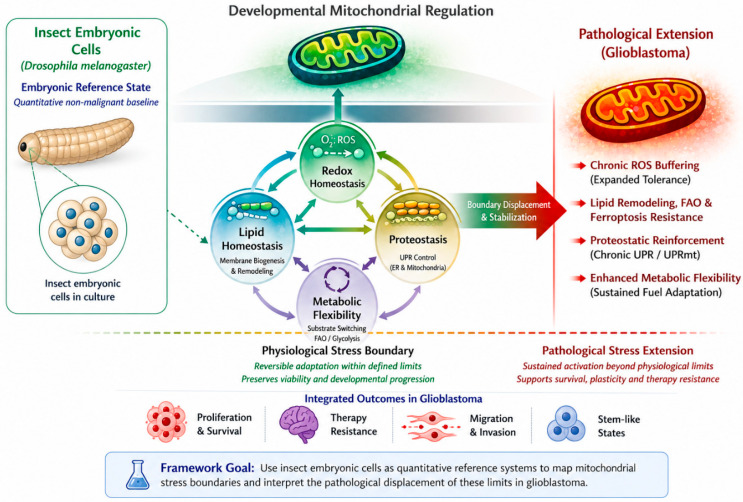
Developmental mitochondrial regulation defines a physiological stress boundary.

These dimensions are mechanistically coupled: oxidative flux influences lipid peroxidation kinetics, lipid composition modulates membrane function and proteostatic load, and proteostasis networks regulate mitochondrial quality control and ROS output [20]. Developmental stress tolerance is therefore proposed to emerge from coordinated mitochondrial regulation across interdependent adaptive systems rather than from any single pathway. This concept provides the biological basis for interpreting GBM stress tolerance as multidimensional boundary displacement rather than isolated metabolic rewiring (Figure 1).

During embryogenesis, mitochondrial coordination of redox balance, lipid homeostasis, proteostasis/UPR, and metabolic flexibility maintains cellular adaptation within a physiological stress boundary—the limit of reversible adaptation. These four axes are functionally coupled, so tolerance emerges from multidimensional constraints, not a single parameter. *Drosophila* embryonic cells provide the proposed non-malignant baseline for this coordinate space. Elements shown are conceptual, not mechanistic.

## 3. Insect Embryonic Systems as Reductionist Platforms for Quantitative Baseline Mapping

Operationalizing the proposed developmental stress boundary framework requires a non-malignant reference system in which mitochondrial stress tolerance can be quantitatively mapped under controlled conditions. Among the available options, including mammalian embryonic stem cells, early murine embryos, *C. elegans*, and primary human cell systems, insect embryonic models, particularly *Drosophila melanogaster*, offer a favorable combination of evolutionary conservation, experimental tractability, and naturally occurring developmental states characterized by high metabolic and biosynthetic demand.

### 3.1. Evolutionary Conservation of Core Mitochondrial and Stress-Response Pathways

Mitochondrial biochemistry is among the most deeply conserved features of eukaryotic life, with core mechanisms of oxidative phosphorylation, ROS generation and detoxification, substrate utilization, and quality control retained across phylogeny [21,22]. Chowdhary et al. [21] characterized mitochondrial organization, respiratory activity, and responses to metabolic perturbation in *Drosophila* blastoderm embryos, identifying features broadly comparable to those described in mammalian developmental systems.

Conservation extends to stress-response pathways relevant to this framework. Ferroptosis-like cell death, an iron-dependent process driven by lipid peroxidation and initially characterized in mammalian cancer cells [18,19], has recently been described in *Drosophila*. Davidson et al. [23] identified lipid peroxidation, iron dependence, and ferrostatin-1-mediated rescue during larval inflammatory responses. Whether this process constitutes ferroptosis in the strict mammalian sense remains debated because insect lipidomes differ substantially from mammalian lipidomes. Mammalian-defining substrates such as arachidonoyl-phosphatidylethanolamine species have not been confirmed in flies, and the functional equivalence of the *Drosophila* GPX4 ortholog has not been conclusively established. Nevertheless, the involvement of iron-dependent lipid peroxidation and sensitivity to ferrostatin-1 suggests partial conservation of the underlying death pathway. This supports the use of insect systems to investigate conserved principles of lipid peroxidation-driven cell death without assuming mechanistic identity with mammalian ferroptosis.

ER- and mitochondria-associated unfolded protein responses are also represented in *Drosophila* by orthologous transcription factors, chaperones, and quality-control pathways [13,24]. More broadly, fundamental stress-response mechanisms can remain conserved even when their regulatory architecture diverges between species [25]. Stress boundaries mapped in insect embryos may therefore reflect broadly conserved bioenergetic constraints rather than exclusively lineage-specific phenomena.

### 3.2. Practical Advantages Relative to Mammalian Embryonic Stem Cells

Mammalian embryonic stem cell systems also have limitations as quantitative reference platforms. Under commonly used culture conditions, particularly LIF/2i-based maintenance of mouse embryonic stem cells, metabolic states may be experimentally stabilized toward glycolytic dependence and reduced oxidative phosphorylation [8,9], potentially influencing derived stress-response thresholds. Insect embryos, by contrast, undergo naturally occurring metabolic transitions during development. Tennessen et al. [26] described a developmentally regulated metabolic switch supporting larval growth, whereas Chowdhary et al. [27] demonstrated dynamic regulation of mitochondrial morphology and activity during cellularization under high biosynthetic demand.

Insect embryos also sustain high metabolic and biosynthetic demand while remaining developmentally viable, providing physiological states in which mitochondrial adaptation can be examined under substantial endogenous load. These features make them potentially useful for mapping the upper range of reversible stress adaptation.

Human neural organoids and GBM organoid models can recapitulate important aspects of brain development and tumor heterogeneity, but their quantitative use is limited by variability, technical complexity, and incomplete standardization [28]. In GBM organoids, retained intratumoral heterogeneity may further complicate the derivation of reproducible stress thresholds [28]. Insect embryonic systems, by contrast, offer synchronized developmental staging, genetic tractability, experimental reproducibility, and direct access to mitochondrial function. These features support their use as calibratable non-malignant reference systems rather than as surrogates of the tumor microenvironment.

### 3.3. Practical Advantages Relative to C. elegans

*C. elegans* is a powerful genetic model, but *Drosophila* offers several features that may be particularly useful for investigating lipid-dependent stress responses. The *Drosophila* fat body integrates liver- and adipose-like functions, including lipid storage, mobilization, and metabolic signaling [11,12], providing a well-developed experimental context for studying how lipid composition influences susceptibility to peroxidative stress. This is relevant to ferroptosis-related mechanisms, in which phospholipid composition and polyunsaturated fatty acid availability are major determinants of lipid peroxidation sensitivity [18,19].

*Drosophila* embryonic systems also provide direct access to cells and tissues suitable for live-cell imaging, experimental dissociation, and scalable biochemical sampling [17,29,30]. These practical features facilitate the integration of imaging with lipidomic and proteomic analyses when constructing quantitative stress-response landscapes.

### 3.4. Experimental Tractability: Primary Culture and Genetic Manipulation

Established protocols for embryonic dissociation, primary culture, and ex vivo manipulation are available [29,30], enabling graded oxidative, metabolic, lipid-peroxidation, and proteotoxic challenges under controlled conditions that are difficult to perform systematically in intact mammalian embryos. Genetic manipulation is supported by advanced tools, including CRISPR/Cas9, RNA interference, and tissue- or stage-specific GAL4/UAS systems, permitting targeted perturbation of GPX4-associated protection against lipid peroxidation, NRF2-ortholog signaling, mitochondrial chaperones, fatty acid oxidation enzymes, and mitophagy regulators in a non-malignant developmental context. Insect systems also permit extensive iterative experimentation under substantially fewer ethical and regulatory constraints than mammalian embryonic models, while their relatively low cost facilitates large-scale dose–response studies.

A practical constraint deserves acknowledgment: many genes involved in the processes central to this framework, including mitochondrial function, redox homeostasis, and proteostasis, are essential for embryonic viability, and null or strong hypomorphic alleles may cause early lethality, precluding analysis at later developmental stages. This can be mitigated using tools already available in the *Drosophila* genetic toolkit rather than constitutive null alleles: temperature-sensitive alleles, GAL80ts and Q-system approaches for temporally restricted perturbation, stage-specific GAL4 drivers, graded RNA interference to reduce activity below the lethal threshold, and ex vivo culture of dissociated cells or tissues to bypass organismal viability constraints.

### 3.5. Insect Embryos as Quantitative Reference Platforms, Not Tumor Surrogates

Insect embryonic systems are not proposed as biological surrogates of glioblastoma. They do not reproduce the genetic lesions, tissue architecture, immune microenvironment, or therapeutic selection pressures that shape GBM evolution in the mammalian brain. Their value lies in providing experimentally tractable, non-malignant systems in which conserved mitochondrial stress-response pathways can be systematically perturbed and their functional outputs quantitatively characterized. Relevant measurements could include redox-buffering capacity, resistance to lipid peroxidation, proteostatic tolerance, metabolic flexibility, and recovery kinetics, together defining comparative developmental baselines. This approach follows the broader logic of reductionist biology, in which conserved mechanisms are dissected in experimentally accessible systems before being validated in more complex pathological contexts [25].

## 4. Experimental Mapping of Developmental Stress Limits

Because insect embryonic systems provide experimentally tractable, non-malignant reference platforms, the proposed developmental stress boundary can be approached quantitatively rather than inferred conceptually. Defining a physiological stress boundary requires quantitative experimental approaches. In insect embryonic systems, mitochondrial stress tolerance can be mapped through:

- Graded oxidative challenge with quantitative viability assessment;
- Quantification of lipid peroxidation thresholds under controlled metabolic load;
- Time-dependent activation kinetics of mitochondrial and ER unfolded protein responses;
- Post-stress recovery assays distinguishing reversible adaptation from irreversible damage [31].

The goal is not to induce irreversible failure but to delineate the physiological range over which adaptation remains reversible. This enables construction of quantitative stress-response landscapes in which viability, recovery, and pathway activation are analyzed as continuous functions of perturbation intensity.

### Toward Quantification of Stress Boundary Displacement

**Figure 2:**
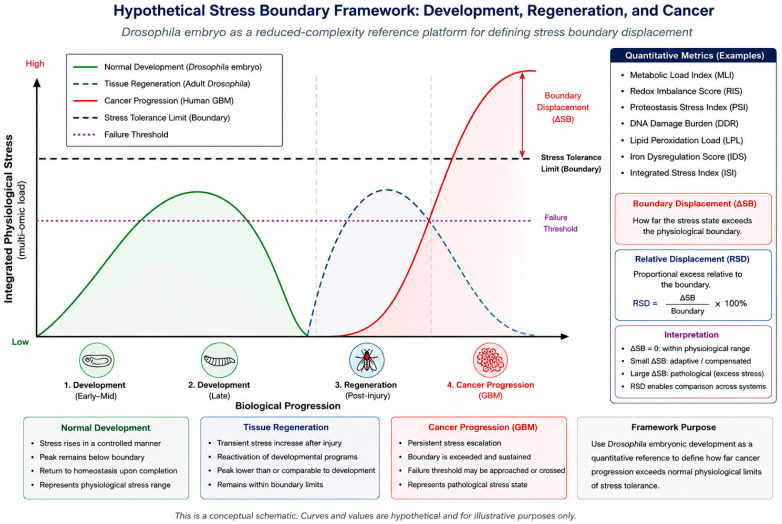
Hypothetical stress boundary framework: development, regeneration, and cancer progression.

Within the proposed framework, “stress boundary displacement” may be operationalized as the quantitative shift between developmental and malignant systems across multiple coupled dimensions of mitochondrial adaptation. These dimensions define a multidimensional state space encompassing redox balance, lipid stability, proteostatic buffering, metabolic flexibility, and recovery capacity. Rather than relying on binary survival endpoints, this state space would be parameterized using continuous stress-response functions. Illustrative candidate descriptors include ROS50 (oxidative challenge producing 50% loss of viability), LPO50 (lipid peroxidation associated with irreversible membrane damage or ferroptotic transition), UPR persistence time, recovery half-time, and stress–response area under the curve (AUC). These descriptors can be summarized into two composite, schematic indices illustrated in Figure 2: boundary displacement (ΔSB), representing the integrated excess of a stress-response trajectory above the physiological boundary over the interval in which it is exceeded, and relative displacement (RSD), which expresses ΔSB as a percentage of the boundary value itself (RSD = ΔSB/Boundary × 100%). Because RSD is dimensionless, it is intended, in principle, to allow comparison of displacement magnitude across otherwise incommensurable stress dimensions (e.g., redox versus proteostatic); this composite framing is illustrative rather than empirically derived, and its utility would need to be established experimentally. In practice, the proposed boundary could be mapped using ROS dose–response viability curves (EC50, slope), recovery of mitochondrial membrane potential after standardized oxidative challenge [32], GPX4-associated lipid peroxidation thresholds under graded hydroperoxide exposure [18,19], UPRmt induction kinetics [24,33], and recovery time following ATP-depleting stress.

The proposed displacement is not expected to be static: stress boundaries are likely to vary across developmental stages, cellular states, nutrient conditions, and microenvironmental contexts, with mesenchymal-like GBM states potentially showing greater displacement than proneural states, and therapeutic pressure potentially inducing transient boundary shifts. Accordingly, the framework describes a dynamic landscape of adaptive constraints rather than a fixed universal threshold.

## 5. Stress Tolerance Displacement: Evidence from Comparative Literature

The hypothesis that glioblastoma extends developmental stress tolerance mechanisms requires comparative support. Direct quantitative comparisons between insect embryonic systems and GBM cells under matched experimental conditions have not yet been performed. Nevertheless, the available literature documents consistent patterns of stress adaptation in developmental and malignant contexts that provide a biologically plausible rationale for the proposed framework.

### 5.1. Oxidative Stress Tolerance: Qualitative Displacement in Malignancy

Mitochondrial ROS serve dual roles: as signaling molecules during normal development and as cytotoxic agents at excessive levels. Bigarella et al. [4] and Sena and Chandel [6] showed that stem cell fate decisions depend on tightly regulated ROS signaling. When ROS levels exceed the physiological signaling range, embryonic and stem cell systems undergo growth arrest, differentiation, or cell death, illustrating the existence of biologically meaningful oxidative limits.

In glioblastoma, oxidative stress tolerance is shifted toward higher stress resilience through antioxidant enzyme upregulation, enhanced NADPH generation, and glutathione-supporting metabolic rewiring [34,35]; NRF2 sustains glioma stem cell maintenance and chemoresistance [36]; and tumor mitochondrial redox signaling operates under conditions incompatible with non-transformed cell viability [37]. Rather than eliminating ROS toxicity, GBM appears to extend the range over which oxidative stress remains compatible with proliferation and survival—the malignant counterpart of the signaling-relevant range described above for developmental and stem cell systems [4,6]. This displacement is not uniform across GBM states: mesenchymal-like states, diverging most from developmental lineage programs, exhibit greater oxidative stress resistance than proneural states [2,3,38], consistent with stress tolerance correlating inversely with proximity to developmental baseline programs.

### 5.2. Lipid Peroxidation and Ferroptosis: Conserved Mechanisms, Shifted Thresholds

Ferroptosis is characterized by the accumulation of lipid hydroperoxides in membranes enriched in polyunsaturated fatty acids (PUFAs), with GPX4 acting as the principal suppressor by reducing lipid peroxides to non-toxic alcohols [18]; it was originally described in cancer cells treated with erastin or RSL3 [19].

Ferroptosis is not confined to malignancy. Davidson et al. [23] recently documented ferroptosis-like cell death in *Drosophila* during inflammatory responses, supporting the view that core biochemical features—including iron-dependent lipid peroxidation, GPX4-mediated protection, and ferrostatin-1 sensitivity—are at least partly conserved and functional in non-malignant developmental contexts, notwithstanding the lipidome differences noted above. This finding strengthens the rationale for using insect systems as platforms for studying lipid peroxidation thresholds under physiological conditions.

In GBM, ferroptosis sensitivity varies across cellular states: mesenchymal-like GBM cells exhibit higher GPX4 expression and greater resistance relative to proneural/intermediate states [39], mechanistically linked to increased cystine uptake via xCT (SLC7A11) and sustained glutathione synthesis [18,39]. In addition, GBM cells engage FAO to support bioenergetics and NADPH production essential for glutathione regeneration and GPX4 activity [40,41].

Within the proposed framework, this pattern is interpreted as quantitative amplification of conserved protective pathways: the lethal threshold is shifted, whereas the underlying biochemical architecture remains conserved across developmental and malignant contexts [18,19,23,39]. Consistent with this interpretation, ferroptosis inducers have shown synergistic effects when combined with standard-of-care therapies in preclinical GBM models [42].

### 5.3. Proteostatic Stress and the Unfolded Protein Response

Cellular proteostasis is maintained through chaperones, the ubiquitin-proteasome system, autophagy, and organelle-specific UPR in the ER and mitochondria [13,14]. During development, these pathways are transiently activated by biosynthetic demand and resolve once homeostasis is restored [13,15]; the UPR can promote either adaptive survival or cell death depending on stress intensity and duration [13].

In glioblastoma, proteostatic responses are chronically active rather than transiently induced: UPRmt is constitutively upregulated in most GBM samples and correlates with chemo/radiotherapy resistance, with elevated HSP60 and ATF5 expression coordinating mitochondrial proteostasis under stress [33]. Cancer cells more broadly exploit proteostasis networks, including the ubiquitin-proteasome system, to tolerate the misfolding burden of rapid proliferation, aneuploidy, and metabolic dysregulation [43]. GBM cells thus appear to maintain proteostatic function under conditions that would compromise it in less stress-tolerant cells, consistent with the sustained, non-resolving character of this reinforcement rather than novel pathway biochemistry [13,14,15,33,43]; UPRmt components including HSP60 and ATF5 are actionable targets in brain tumors [44].

### 5.4. Metabolic Flexibility and Substrate Utilization

Metabolic adaptability characterizes both development and malignancy [8,9,10,45]. During development, cells transition between glycolytic and oxidative metabolism in response to nutrient availability, oxygen tension, and differentiation signals [8,9]; reprogramming to pluripotency requires a reversible, tightly regulated shift from oxidative phosphorylation to glycolysis [8], and stem cell fate decisions are coupled to mitochondrial dynamics [9].

Cancer cells deploy similar flexibility in a sustained, often exaggerated manner, rewiring central carbon metabolism to support biosynthesis, redox homeostasis, and ATP production under nutrient-limited/hypoxic conditions [45]. In GBM, cells upregulate FAO to sustain proliferation under glucose deprivation and generate NADPH for antioxidant defense [41], functionally integrated with redox and proteostatic adaptation via FAO-supported glutathione regeneration and GPX4 activity [40,41]. Rather than introducing fundamentally novel metabolic pathways, these adaptations appear to expand the physiological range over which conserved mitochondrial programs remain functional [8,9,41,45]. Together, these observations indicate that metabolic plasticity is another dimension along which developmental stress boundaries may become displaced during malignant transformation.

### 5.5. Synthesis: Amplification of Conserved Pathways, Not Invention of Novel Mechanisms

Across the four dimensions examined—redox buffering [4,6,34,35,36,37], lipid peroxidation resistance [18,19,23,39], proteostatic reinforcement [13,14,33,43], and metabolic flexibility [8,9,41,45]—GBM cells are proposed not to invent fundamentally new biochemical mechanisms, but to extend, stabilize, and amplify existing adaptive programs beyond the constraints of developmental physiology. This forms the biological rationale for the framework and yields a testable prediction: interventions compressing stress tolerance toward developmental limits (NRF2 inhibition [34], GPX4 degradation [39], proteostatic disruption [33,43], FAO blockade [41]) should restore therapeutic sensitivity in proportion to threshold displacement—a magnitude that cannot yet be precisely quantified without the comparative experiments this framework motivates.

These dimensions are also mechanistically coupled (ROS flux shapes lipid peroxidation; lipid composition feeds back on membrane function and proteostatic load; proteostatic machinery regulates mitochondrial quality and ROS output; metabolic substrate selection influences all three [4,5,6,7,13,19,20,34,35,36,37,39,40,41,42,43,44,45,46]), consistent with emerging frameworks treating mitochondrial cancer biology as coupled adaptive modules. This coupling implies that single-node targeting may trigger compensatory flux through alternative pathways and that effective intervention will likely require simultaneous compression of multiple dimensions. Although direct quantitative comparisons with developmental systems remain unavailable, the convergence of these observations across all four dimensions provides a coherent biological rationale for experimentally testing the proposed framework.

## 6. Conceptual Integration and Existing Gap

Mitochondrial metabolism, redox signaling, lipid remodeling, and proteostasis have each been extensively studied in embryogenesis and cancer biology [2,3,4,5,6,7,8,9,10,11,12,13,14,15,16,17,18,19,20,34,35,36,37,38,39,40,41,42,43,44,45], and GBM plasticity has been linked to metabolic adaptation, mitochondrial reprogramming, and transcriptional states resembling embryonic progenitors [2,3,47,48].

Existing frameworks connecting development and cancer—the cancer stem cell hypothesis, EMT models, oncofetal gene reactivation—predominantly ask which developmental programs are reactivated in malignancy [47,48]. This framework instead asks what physiological limits constrain these programs normally and to what extent malignant cells exceed them—a shift from a descriptive to a metric perspective requiring a non-malignant reference system, a role insect embryonic systems are positioned to fill. Within this perspective, malignant adaptation becomes a quantitative problem rather than a categorical one. Here, “stress boundary” denotes the limit of reversible adaptation and “threshold” the point at which it is exceeded; because adaptive capacity is state-dependent, the boundary is a dynamic constraint rather than a single immutable value. Table 1 summarizes key features of mitochondrial stress tolerance across developmental and malignant contexts.

Table 1 separates literature-supported observations from the conceptual interpretations proposed here; the cited studies document individual biological features, not a direct, matched-condition comparison between insect embryos and GBM cells. This distinction motivates the experimental agenda that follows. The next section applies the framework to glioblastoma by organizing mitochondrial stress adaptation into four interrelated functional modules.

To make this novelty explicit, Table 2 situates the proposed framework relative to established concepts it builds on.

This table is intended to clarify contribution rather than to claim exhaustiveness; existing models remain necessary for aspects of GBM biology—genetic, immune, microenvironmental—that lie outside the scope of a stress-tolerance metric.

### Tissue Regeneration as a Complementary Physiological Reference Point

Adult tissue regeneration after injury shares features with both development and malignant growth—rapid proliferation, metabolic adaptation, and reactivation of developmental transcriptional programs in a post-developmental context [9,50,51]—but remains self-limited where tumor progression does not, making the comparison useful for isolating what distinguishes pathological from physiological stress tolerance.

Within the proposed framework, regeneration is interpreted as stress transiently elevated above baseline that remains within, or returns to, the physiological stress boundary once repair is complete, unlike GBM, where stress tolerance is hypothesized to be persistently displaced beyond it (Figure 2). This distinction is conceptual, not quantitatively established: the trajectories in Figure 2 are a hypothesis-generating illustration, not derived data. Adult *Drosophila* regeneration paradigms (e.g., imaginal disc or gut regeneration) could serve as an additional non-malignant, post-developmental reference point, distinguishing “elevated but bounded” stress tolerance (regeneration) from “elevated and unbounded” tolerance (malignancy).

This conceptual schematic depicts hypothesized—not measured—stress trajectories across three contexts: normal *Drosophila* development, adult tissue regeneration after injury, and GBM progression, plotted against a common stress tolerance limit and failure threshold. Development and regeneration remain within the boundary; GBM is depicted as exceeding it, illustrating “boundary displacement” (Section Toward Quantification of Stress Boundary Displacement). Curve shapes and values are illustrative only and do not represent experimental measurements.

## 7. Mitochondrial Stress Adaptation in Glioblastoma

**Figure 3:**
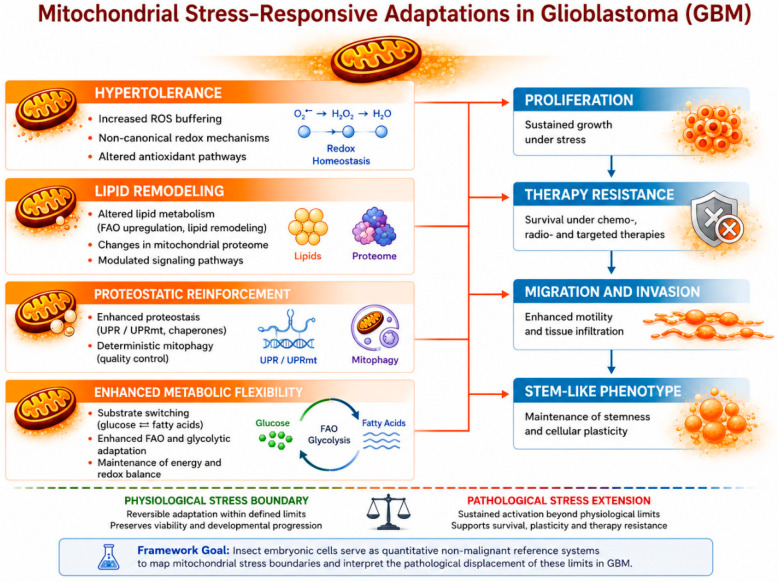
Mitochondrial stress adaptations in glioblastoma extend developmental limits.

GBM cells are characterized by a remarkable capacity to tolerate elevated oxidative and metabolic stress [33,34,35,36,37,39,41,43,45], with altered lipid metabolism and ferroptosis-associated vulnerabilities [18,39], enhanced FAO [40], reinforced proteostatic buffering [14,33], and highly dynamic cellular states [2,3]. Within the developmental stress boundary framework, these adaptations can be organized into four interrelated functional modules (Figure 3): hypertolerance (expanded ROS buffering), lipid remodeling (FAO upregulation, ferroptosis-like resistance), proteostatic reinforcement (chronic UPR/UPRmt activation, selective mitophagy [33,44,46]), and enhanced metabolic flexibility, which enables the other three via NADPH regeneration and membrane lipid supply. This organization is a conceptual framework for interpretation, not a rigid biological classification.

GBM cells are proposed to sustain mitochondrial stress adaptation across the four modules above. Phenotypic outputs—proliferation, therapy resistance, migration, stem-like states—are thought to emerge from these integrated programs. The figure summarizes hypothesized trends, not measured or mechanistic sequences.

Collectively, these four modules are proposed to reflect sustained activation beyond the physiological stress boundary defined during development (Figure 1): the tumor, on this view, does not rewrite the architecture of cellular stress responses but shifts their position and expands their stability, so that GBM’s stress-tolerance landscape is best understood as a broadened, stabilized version of the adaptive space embryonic cells traverse transiently.

## 8. Limitations and Competing Models

The framework has important limitations. Insect embryonic systems are proposed as quantitative reference platforms, not biological surrogates: they do not recapitulate the genetic lesions, tissue architecture, immune microenvironment, or therapeutic selection pressures governing GBM evolution in the mammalian brain, and the value of the comparison lies in experimental tractability rather than biological equivalence. The model is also deliberately boundary-centered and may underrepresent qualitative innovations of malignant progression: some GBM adaptations likely reflect extension of physiological stress programs and others may arise through oncogene-driven rewiring, chromatin remodeling, or microenvironmentally induced transitions without a developmental equivalent.

Several established models compete with and complement this interpretation: the glioma stem cell model emphasizes tumor-propagating populations and hierarchical organization [47,49]; single-cell analyses show GBM recapitulating neural developmental hierarchies across interchangeable states [2,52]; mesenchymal transition models attribute aggressive behavior to microenvironmentally driven state switching [38,53]; and the metabolic reprogramming framework treats fuel use and redox buffering as proximal survival determinants without reference to developmental boundary displacement [40,45]. These models are not mutually exclusive—each addresses a different level of GBM biology—and the developmental stress boundary framework is intended to complement, rather than replace, them by adding a quantitative developmental reference. Its value will depend on whether developmental baselines improve mechanistic interpretation and therapeutic prediction beyond current GBM-centered models.

## 9. Discussion and Conclusions

If the developmental stress boundary framework is valid, GBM-derived cells should exceed, but not fundamentally differ from, the stress-response architecture of insect embryonic systems under matched perturbation conditions: dose–response curves for ROS-induced viability loss, lipid peroxidation, and UPR activation should be right-shifted in GBM lines without alteration in response shape. GBM lines with closer transcriptional similarity to developmental progenitors [2,3] should display stress tolerance profiles nearer the embryonic baseline, while mesenchymal-state cells diverging most [38,52] should show the greatest threshold displacement. Pharmacological interventions compressing stress tolerance toward developmental limits—inhibiting FAO [40] or proteostatic buffering [33]—would be expected to restore ferroptotic/proteotoxic sensitivity in proportion to displacement. Taken together, these predictions turn a conceptual claim into a testable research program: establishing developmental baselines against which GBM resilience, and rational combination therapy, can be measured rather than assumed.

### 9.1. Future Research Directions Arising from the Framework

The following points are offered as future research directions rather than as clinical implications: they describe hypotheses that would need to be tested before any bearing on therapeutic decision-making could be considered, and none of them should be read as a basis for current clinical practice. If experimentally supported, the developmental stress boundary framework could carry implications for GBM therapy, arising from the hypothesis that malignant stress tolerance reflects amplification of conserved developmental programs. Comparative studies using adult *Drosophila* regeneration paradigms (Section Tissue Regeneration as a Complementary Physiological Reference Point) alongside embryonic baselines could further test whether the stress displacement observed in GBM is specific to malignancy or shared more broadly with other instances of accelerated, developmentally reactivated growth.

### 9.2. Prediction 1: Cellular State May Predict Stress Tolerance and Therapy Response

GBM cells occupy multiple interconvertible states—proneural, neural, astrocytic, and mesenchymal-like—with variable developmental transcription factor and metabolic pathway expression, plastic under microenvironmental, genetic, and therapeutic pressure [2,3]. States retaining greater similarity to developmental programs should exhibit stress tolerance closer to the embryonic baseline, whereas mesenchymal-like cells should show the greatest displacement—consistent with elevated GPX4 expression and ferroptosis resistance in mesenchymal versus proneural GBM cells [39] and microenvironmentally driven, therapy-resistant mesenchymal differentiation [38]. Patient stratification by cellular-state composition might therefore predict responsiveness to stress-tolerance-targeting therapies: proneural-enriched tumors to ferroptosis inducers and mesenchymal-enriched tumors to more aggressive multi-modal compression [2,38,39,52].

### 9.3. Prediction 2: Combination Therapy via Stress Threshold Compression

Standard-of-care therapy leaves median overall survival at ~15 months [1], possibly because current therapies fail to compress stress tolerance beyond adaptive capacity. Simultaneous targeting of redox buffering, lipid peroxidation resistance, proteostatic reinforcement, and metabolic flexibility may be needed to prevent compensatory adaptation [34,39,41,42,43], with efficacy expected to scale with baseline displacement.

### 9.4. Prediction 3: Developmental Reference Points May Identify Therapeutic Windows

If malignant stress tolerance reflects extension of developmental programs, specific developmental transitions may reveal transient vulnerabilities informing therapeutic timing. *Drosophila* cellularization, for instance, involves mitochondrial fragmentation under high biosynthetic demand [27]—a potential analogue for vulnerability during GBM mitosis, migration, or metabolic switching, consistent with chronotherapy rationale [1,54]. GBM cells undergo metabolic rewiring within hours of nutrient stress [41]; targeting this transition before compensatory FAO upregulation may offer a clinically relevant window.

### 9.5. Prediction 4: Monitoring Stress Tolerance Compression as a Biomarker

Biomarkers reflecting stress-buffering capacity—circulating lipid peroxidation products, GSSG/GSH ratios, and circulating mitochondrial DNA—could be explored as candidate pharmacodynamic markers, where a post-therapy decrease could inform patient stratification and timing of subsequent stress-compressing interventions.

### 9.6. Limitations and Alternative Interpretations

Not all GBM resistance mechanisms reduce to stress tolerance displacement. Genetic heterogeneity, tumor microenvironment interactions, immune evasion, and blood–brain barrier penetration all contribute in ways that may be orthogonal to mitochondrial stress tolerance [1,3,38], and some adaptations—MGMT-mediated DNA repair after temozolomide, for instance—are qualitatively distinct, with no developmental equivalent. The developmental stress boundary framework is therefore best read not as a comprehensive account of GBM resistance, but as one specific, testable hypothesis about its metabolic and oxidative basis—one whose value will ultimately rest on whether developmental baselines sharpen the prediction of therapeutic response beyond what current GBM-centered models already offer.

Viewed from this perspective, glioblastoma may be understood not as a system that invents fundamentally new stress-response mechanisms, but as one that progressively displaces the operational limits of evolutionarily conserved adaptive programs. Quantifying this displacement, beginning with the developmental baselines proposed here, may offer a new experimental language for interpreting malignant stress tolerance.

## Figures and Tables

**Table 1:** Literature-supported observations and conceptual inferences underlying the developmental stress boundary framework.

Dimension	Literature-Supported Observations	Conceptual Interpretation in this Framework
Biological context	Insect embryonic systems are non-malignant developmental models; glioblastoma is genetically heterogeneous and cell-state plastic [2,3,47,48].	Developmental systems can serve as non-malignant reference baselines rather than tumor surrogates.
ROS regulation	Mitochondrial ROS act as signaling mediators but can become damaging when excessive [4,5,6,7]. Cancer cells frequently adapt to elevated oxidative stress [34,35,36,37].	GBM may represent a pathological shift in the tolerated range of redox stress.
Lipid metabolism	Insect development requires coordinated lipid metabolism and membrane biogenesis [11,12]. GBM shows lipid metabolic rewiring, including FAO and ferroptosis-related vulnerabilities [18,39,40,41].	Lipid stress tolerance may be broader or more persistently buffered in GBM than in development.
Proteostasis/UPR	Developmental and cellular stress responses require proteostatic control [13,14,15]. Cancer cells can reinforce proteostasis and mitochondrial stress responses [33,43].	Sustained proteostatic reinforcement may help GBM remain viable beyond physiological stress limits.
Mitochondrial developmental dynamics	Mitochondrial organization, activity, and metabolic transitions are important during *Drosophila* embryogenesis [26,27].	Insect embryos may allow experimental mapping of mitochondrial stress-response boundaries.
Stress tolerance thresholds	The literature supports stress-dependent transitions between adaptation and damage, but direct insect embryo–GBM threshold comparisons are not yet available [4,7,13,14,34,35,36,37].	The proposed “stress boundary” is a testable hypothesis, not an established comparative measurement.
Recovery capacity	Recovery after stress is discussed in the proteostasis and stress-response literature, but direct cross-system recovery comparisons remain limited [13,14,33,43].	Recovery curves could be used experimentally to distinguish reversible adaptation from irreversible collapse.
Functional outcome	Embryonic systems support developmental progression, whereas GBM plasticity supports tumor persistence and progression [2,3,47,48,49].	Malignant stress tolerance may be interpreted as stabilization of adaptive programs normally constrained during development.

**Table 2:** Positioning the developmental stress boundary framework relative to existing concepts.

Existing Concept	Addressed by the Prior Literature	Addressed by This Framework
Metabolic plasticity/rewiring	Yes [8,9,10,40,41,45]	Incorporated as one of four coupled adaptive dimensions
Cancer stem cell/stemness models	Yes [47,48,49]	Referenced as a complementary, state-based model
Stress adaptation (redox, proteostatic)	Yes [4,5,6,7,13,14,15,33,34,35,36,37,43]	Incorporated as coupled adaptive dimensions
A shared, non-malignant quantitative developmental baseline	Not established	Proposed (insect embryonic reference platform)
An explicit metric for “how far beyond development” (boundary displacement)	Not established	Proposed (Section Toward Quantification of Stress Boundary Displacement)
Regeneration as a bounded physiological comparator to malignancy	Discussed separately in the regeneration literature [9,50,51]	Proposed integration with the same boundary concept (Section Tissue Regeneration as a Complementary Physiological Reference Point)

## Data Availability

No new data were created or analyzed in this study. Data sharing is not applicable to this article.

## Abbreviations

GBM	glioblastoma
ROS	reactive oxygen species
UPR	unfolded protein response
UPRmt	mitochondrial unfolded protein response
ER	endoplasmic reticulum
FAO	fatty acid oxidation
ESCs	embryonic stem cells
GPX4	glutathione peroxidase 4
GSSG	glutathione disulfide
GSH	reduced glutathione
NRF2	nuclear factor erythroid 2-related factor 2
NADPH	nicotinamide adenine dinucleotide phosphate
HSP60	heat shock protein 60
ATF5	activating transcription factor 5
